# Low risk of neurodevelopmental impairment among perinatally acquired HIV‐infected preschool children who received early antiretroviral treatment in Thailand

**DOI:** 10.1002/jia2.25278

**Published:** 2019-04-16

**Authors:** Watsamon Jantarabenjakul, Weerasak Chonchaiya, Thanyawee Puthanakit, Tuangtip Theerawit, Jesdaporn Payapanon, Jiratchaya Sophonphan, Montida Veeravigom, Neda Jahanshad, Paul M Thompson, Jintanat Ananworanich, Kathleen Malee, Chitsanu Pancharoen, the DOET study

**Affiliations:** ^1^ Department of Pediatrics Faculty of Medicine Chulalongkorn University Bangkok Thailand; ^2^ Center of Excellence in Pediatric Infectious Diseases and Vaccines Faculty of Medicine Chulalongkorn University Bangkok Thailand; ^3^ Thai Red Cross Emerging Infectious Diseases Clinical Center King Chulalongkorn Memorial Hospital Bangkok Thailand; ^4^ Maximizing Thai Children's Developmental Potential Research Unit Faculty of Medicine Chulalongkorn University Bangkok Thailand; ^5^ The HIV Netherlands Australia Thailand Research Collaboration (HIV‐NAT) Thai Red Cross AIDS Research Centre Bangkok Thailand; ^6^ Imaging Genetics Center Stevens Neuroimaging and Informatics Institute Keck School of Medicine of USC Los Angeles CA USA; ^7^ SEARCH, The Thai Red Cross AIDS Research Center (TRCARC) Bangkok Thailand; ^8^ Henry M. Jackson Foundation for the Advancement of Military Medicine Bethesda MD USA; ^9^ Department of Global Health The University of Amsterdam Amsterdam The Netherlands; ^10^ Ann & Robert H. Lurie Children's Hospital of Chicago Northwestern University Feinberg School of Medicine Chicago IL USA

**Keywords:** early treated HIV children, neurodevelopmental outcome, global developmental impairment, PHIV children, PHEU children, Mullen Scales of Early Learning

## Abstract

**Introduction:**

Antiretroviral therapy (ART) is recommended in perinatally HIV‐infected (PHIV) infants immediately upon diagnosis. We aimed to compare neurodevelopmental outcomes between PHIV children who initiated ART within 12 months of life and perinatally HIV‐exposed uninfected (PHEU) children and to assess neurodevelopmental outcomes by timing of ART.

**Methods:**

This prospective cohort study included Thai children aged 12 to 56 months who were assessed with the Mullen Scales of Early Learning (MSEL) at enrolment and at 48 weeks. Global Developmental Impairment (GDI) was defined as Early Learning Composite (ELC) ≤ 70 on the MSEL; typical developmental pattern was defined as ELC > 70 at both visits. Logistic regression was used to compare prevalence of any GDI. Predictors of changing ELC scores were analysed with generalized estimating equations linear regression model.

**Results:**

From 2016 to 2017, 50 PHIV (twenty‐seven early ART within three months and twenty‐three standard ART within three to twelve months) and 100 PHEU children were enrolled. Median (IQR) age at first assessment was 28 (19 to 41) months. PHIV children had lower age‐relevant Z scores for weight, height and head circumference compared to the PHEU group (*p* < 0.05).

The prevalence of overall GDI was 18% (95% CI 11 to 27) and 32% (95% CI 20 to 47) in PHEU and PHIV children respectively (*p* =* *0.06). In subgroup analysis, 22% (95% CI 9 to 42) of early ART PHIV children and 44% (95% CI 23 to 66) of standard ART PHIV children had overall GDI. There was a higher rate of GDI in standard ART PHIV children (*p *=* *0.01), but not in the early ART group (*p *=* *0.62) when compared with PHEU children. The standard ART PHIV group demonstrated lower typical developmental pattern than both the early ART PHIV group and the PHEU group (57% vs. 77% vs. 82% respectively). Non‐attendance at nursery school was associated with changes in ELC score during study participation (adjusted coefficient −3.8; 95% CI −6.1 to −1.6, *p *=* *0.001).

**Conclusions:**

Preschool children with HIV who initiated ART in the first three months of life had a similar rate of GDI as PHEU children. Lack of nursery school attendance predicted poor developmental trajectory outcomes among PHIV children.

## 1 Introduction

Despite the efficacy of antiretroviral therapy (ART) in reducing mortality in children with perinatally acquired HIV infection (PHIV), neurodevelopmental risk is observed among children who initiate ART later in life. In resource‐rich settings, treated PHIV children without history of AIDS‐defined symptoms often demonstrate near normal developmental functioning in all domains and are comparable to perinatally HIV‐exposed uninfected (PHEU) children 1, 2, 3, 4. However, some studies reveal subtle but significant differences in executive function, language skills and memory in PHIV children as they develop 3, 5, 6. In resource‐limited settings, the rate of global developmental impairment (GDI) in children with PHIV varies, with a prevalence of 52% to 60% in Africa and 20% to 50% in Thailand amongst cohorts with limited or no access to early ART 7, 8, 9, 10, 11, 12. The Pediatric Randomized to Early versus Deferred ART Initiation in Cambodia and Thailand (PREDICT) study of chronically treated children observed significantly lower neurodevelopmental scores in PHIV children compared to those with PHEU 13. The pathogenesis of GDI in PHIV children may include direct effects of HIV on the CNS and indirect effects from systemic illness, nutritional status and psychosocial factors 14, 15, 16. There is likely a limited window of opportunity to mitigate HIV insults to the brain. Nonetheless, ART initiated before the age of one year may prevent or minimize neurological and neurodevelopmental impairment in PHIV individuals 13. Infants treated by three months of age in the Children with HIV Early Antiretroviral Therapy Trial (CHER) in South Africa had superior neurodevelopmental outcomes than PHIV infants who deferred ART 17, 18.

Overall there are scarce data comparing neurodevelopmental outcomes in PHIV and PHEU children from developing countries. Thailand is an upper middle income country with free universal healthcare coverage that reached the World Health Organization's targets for elimination of mother to child transmission of HIV in 2016 19. Since 2010, the Thai National Guideline recommends HIV DNA PCR for early infant diagnosis and immediate ART in those infected regardless of symptoms and CD4 + T cell 20. However, only 83% of infants are treated within the first year of life 21. Careful assessment of precise timing of ART during infancy and neurodevelopmental outcomes could provide tangible results to motivate clinicians and policy makers towards implementing early ART in infants as recommended by treatment guidelines 20, 22.

This study primarily aimed to compare neurodevelopmental outcomes between PHIV children who initiated ART within 12 months of life and PHEU children. A secondary aim was to assess the outcomes by timing of ART initiation before and after three months. We hypothesized that the GDI rates would not be significantly different between PHIV children treated by 12 months of age and PHEU children. Moreover, the GDI rate would be lower in PHIV children treated by three months versus those treated later.

## 2 Methods

### 2.1 Study design and participants

This was a prospective, cohort study of young children aged 12 to 56 months born to mothers with HIV in Thailand between 2016 and 2018. Inclusion criteria for PHIV children were: HIV infection with documented positive HIV DNA PCR and receipt of ART within the first year of life. Early ART PHIV group initiated ART at age ≤3 months. Standard ART PHIV group initiated ART at ages >3 to ≤12 months.

Inclusion criteria for PHEU children were: born to mothers with HIV, no HIV infection documented by negative HIV DNA PCR at age >4 months or non‐reactive anti‐HIV antibody at age >12 months. PHEU children were age‐matched to PHIV children within a range of three to six months. Exclusion criteria were: prematurity (gestational age <34 weeks), major congenital anomalies or genetic disorders, current neurologic diseases, or persistent or active AIDS defining opportunistic infection within 30 days prior to enrolment. This study was approved by the Research Ethics Committee, Faculty of Medicine, Chulalongkorn University, Bangkok, Thailand. Written consent was obtained from legal guardians prior to enrolment.

The PHIV group was recruited from the Thailand register database which identified 180 PHIV children who were born during 2012 to 2016 and initiated ART within one year of age; their primary caregivers were invited to participate in this study. The PHEU group was recruited from King Chulalongkorn Memorial Hospital, Bangkok, which provides care for 200 PHEU children who were born during 2012 to 2016.

### 2.2 Neurodevelopmental assessment

Neurodevelopmental outcomes were assessed by the Mullen Scales of Early Learning (MSEL), a standardized comprehensive developmental assessment for children from birth to 68 months of age 23, 24. The MSEL assesses five developmental domains including gross motor, visual reception, fine motor, receptive language and expressive language. Age‐norm T‐scores (commonly used standardized test statistics with a mean of 50 and a standard deviation of 10) were obtained from normative tables in the MSEL administration manual by chronological age (not adjusted age for prematurity). Each domain with T scores ≤30 indicates significant impairment in that domain. The gross motor developmental quotient was calculated separate from other domains due to the age range covered from birth up to only 33 months old. The gross motor developmental quotient (GMDQ) was calculated from the age equivalent score divided by actual age, multiplied by 100, with a mean of 100 and a standard deviation of 15. The GMDQ of ≤70 indicated gross motor developmental impairment. An early learning composite (ELC) score was calculated from total scores of all subscales, with the exception of the gross motor domain. ELC scores have a mean of 100 and standard deviation of 15. ELC scores of ≤70 indicate global developmental impairment (GDI). MSEL was administered using child's age appropriate test activities at enrolment, and at 48 weeks by well‐trained developmental paediatricians who were blinded to the children's HIV status. Each primary caregiver received advice with respect to the child's developmental outcomes and developmental promotion activities specific to each participant's context. Children with significant developmental problems were referred for appropriate diagnostic and therapeutic services.

Trajectory pattern of neurodevelopment was defined by the ELC score at the baseline and 48 week visit and included four categories: (1) Typical GDI: ELC scores >70 at both visits, (2) Resolving GDI: baseline ELC ≤ 70, which improved to >70 at the 48‐week follow‐up, (3) Emerging GDI: baseline ELC > 70 followed by a decline to ELC ≤ 70 at 48 weeks, (4) Persistent GDI: ELC ≤ 70) at both visits.

### 2.3 Clinical and socio‐economic assessment

Primary caregivers provided demographic information regarding children and parent, family history, child‐rearing history and socio‐economic status. Additional clinical information was obtained from the hospital database. Physical examinations, including measurement of weight, height and head circumference, were performed at each visit; raw scores were converted to Z‐scores using the WHO child growth standard reference population with adjustment for prematurity (gestational age 34 to 37 weeks) up to the age of 24 months 25. All children were assessed for anaemic status using WHO criteria (haemoglobin <11 g/dL) at both visits 26. CD4 + T‐cell count and HIV‐RNA were evaluated at each visit in PHIV children only. Depression status of the primary caregiver was assessed by the Thai version of the Patient Health Questionnaire‐9 (PHQ‐9); depression was characterized by a total score of ≥9 27.

### 2.4 Statistical analysis

Characteristics were reported as median and interquartile ranges for continuous variables and percentage for categorical variables. We used the chi‐squared test or the Fisher's exact test to compare categorical variables. The Wilcoxon rank sum test or the Kruskal–Wallis test were used to compare continuous variables. MSEL scores were presented as mean (standard deviation) and compared between PHEU and PHIV children using independent two sample *t* tests. Rate of GDI and their 95% confidence interval (CI) were estimated based on the binomial distribution. The overall rate of GDI included children who had GDI at enrolment and/or follow‐up visit. Odds ratio (OR) and 95% CI for comparing the prevalence of any GDI between PHEU and PHIV (early and standard ART) children was estimated by logistic regression. Generalized estimating equations (GEE) for linear regression were used to analyse predictors of change in ELC scores over time. Multivariate models were developed including covariates with *p* < 0.1 from univariate model. Covariates were demographics, including the children's sex, gestational age, birthweight and exposure to antiretroviral prophylaxis for prevention of mother‐to‐child transmission (PMTCT); family history, including parents’ and caregiver's age, education, maternal history of substance use, child‐rearing history and income; HIV characteristics, including the child's age at ART start, duration of ART, CD4 + T‐cell counts and HIV‐RNA. Statistical significance was defined as *p *<* *0.05. We calculated sample size under the assumption that 24.1% PHIV group developed GDI and 7.6% PHEU group developed GDI 28. The primary objective was to compare a rate of GDI between PHIV and PHEU children. We calculated the sample‐size ratio 1:2, 50 for PHIV and 100 for PHEU children were needed to have 80% power for two‐ sided significance level of 5%. We used STATA software, version 13.1 (Stata Corp., College Station, Texas, USA) for analysis.

## 3 Results

### 3.1 Participants and baseline characteristics

From 2016 to 2017, 50 PHIV children (27 early ART and 23 standard ART) and 100 PHEU children were enrolled. Three PHEU children did not complete the study due to relocation and only had baseline data included. Baseline characteristics of study participants are shown in Table 1. Overall median age (IQR) at enrolment was 29 (22 to 36) months and 27 (19 to 42) months in the PHIV and PHEU groups respectively. PHIV children had significantly lower weight for age, height for age and head circumference for age Z scores when compared with PHEU individuals. There were no statistically significant differences in the rate of anaemia or nursery school attendance among groups.

**Table 1:** Demographic data and clinical characteristics of children

Characteristics	PHEU	All PHIV	Early ART PHIV	Standard ART PHIV	*p* value^a^	*p* value^b^
At the first assessment	n = 100	n = 50	n = 27	n = 23		
Age, months, median (IQR)	27 (19 to 42)	29 (22 to 36)	25 (18 to 30)	35 (28 to 41)	0.20	0.01
Sex: male, n (%)	45 (45%)	28 (56%)	16 (59%)	12 (53%)	0.7	0.43
Low birth weight (birth weight <2500 g), n (%)	18 (18%)	15 (30%)	7 (30%)	8 (30%)	0.14	0.26
Preterm (GA 34 – <37 weeks), n (%)	32 (32%)	19 (38%)	9 (33%)	10 (44%)	0.49	0.59
Weight for age Z‐score, median (IQR)	−0.3 (−0.9 to 0.5)	−0.63 (−1.4 to 0.02)	−0.3 (−1.2 to 0.4)	−0.7 (−1.5 to −0.4)	0.02	0.01
Height for age Z‐score, median (IQR)	−0.6 (−1.3 to 0.1)	−1.1 (−1.6 to −0.4)	−0.8 (−1.6 to −0.2)	−1.3 (−2 to −0.9)	0.005	0.002
Head circumference for age Z‐score, median (IQR)	−0.8 (−1.5 to −0.1)	−1.34 (−2.4 to −0.2)	−0.8 (−2.3 to 0.1)	−1.6 (−2.4 to −0.2)	0.03	0.06
Anaemia (Hb < 11 g/dL), n (%)	14 (14%)	12 (24%)	7 (26%)	5 (22%)	0.13	0.27
No nursery school attendance, n (%)	67 (67%)	34 (68%)	22 (81%)	12 (52%)	0.9	0.09
At 48‐week visit	n = 97	n = 47	n = 27	n = 23		
Weight for age Z‐score, median (IQR)	−0.2 (−0.9 to 0.4)	−0.8 (−1.5 to −0.1)	−0.6 (−1.6 to 0)	−0.8 (−1.3 to −0.3)	0.001	0.003
Height for age Z‐score, median (IQR)	−0.8 (−1.3 to −0.2)	−1.0 (−1.7 to −0.4)	−0.6 (−1.5 to 0)	−1.4 (−2 to −0.7)	0.04	0.03
Head circumference for age Z‐score, median (IQR)	−0.3 (−1 to 0.5)	−0.8 (−1.5 to −0.5)	−0.8 (−1.4 to −0.4)	−0.8 (−1.6 to −0.5)	<0.001	0.002
Anaemia (Hb < 11 g/dL), n (%)	12 (12%)	7 (14%)	5 (19%)	2 (9%)	0.78	0.57
No nursery school attendance, n (%)	52 (54%)	27 (54%)	18 (67%)	9 (39%)	1.0	0.15

Early ART PHIV, PHIV children who early initiated antiretroviral therapy within three months of age; GA, gestational age; PHEU, perinatally HIV‐exposed uninfected children; PHIV, perinatally HIV‐infected children; Standard ART PHIV, PHIV children who initiated antiretroviral therapy within three to twelve months of age.

^a^ *p*‐value between PHEU and PHIV; ^b^ *p‐*value among PHEU, early PHIV and standard PHIV.

The median age (IQR) of ART initiation was 2.1 (1.5 to 2.8) and 5.3 (4.2 to 6.7) months old in the early ART and standard ART PHIV children respectively (Table 2). Among 27 early ART PHIV children, four children were initiated ART during the age of two to four weeks, nine children during >4 to 8 weeks and 14 children during >8 to 12 weeks of age. Among 23 standard ART PHIV children, 15 children were initiated ART during three to six months old and eight children during >6 to 12 months of age. Most PHIV children were CDC class N or A; three children with standard ART PHIV were CDC class C. Overall, 72% of PHIV children were virologically suppressed at the time of developmental assessment. Ten PHIV children were persistently unsuppressed at both visits and eight PHIV children had dynamic changes in virological status between visits.

**Table 2:** Clinical characteristics of perinatally HIV‐infected children (PHIV) children

Characteristics	All PHIV (n = 50)	Early ART PHIV (n = 27)	Standard ART PHIV (n = 23)	*p* value
At the first assessment				
Age started ART, months, median (IQR)	2.9 (1.9 to 5.1)	2.1 (1.5 to 2.8)	5.3 (4.2 to 6.7)	<0.001
Current ART regimen, n (%)				
PI based regimen	42 (84%)	23 (85%)	19 (83%)	0.84
NNRTI‐based regimen	8 (16%)	4 (15%)	4 (17%)	
CD4 + T‐cell count (cells/μL), median (IQR)	1824 (1139 to 2188)	1943 (1370 to 2885)	1725 (1340 to 2363)	0.37
HIV RNA < 200 copies/mL, n (%)	37 (74%)	19 (70%)	18 (78%)	0.53
At 48‐week visit				
CD4 + T‐cell count (cells/μL), median (IQR)	1824 (1139 to 2188)	1570 (1239 to 1818)	1409 (1121 to 1829)	0.42
HIV RNA < 200 copies/mL, n (%)	35 (70%)	19 (70%)	16 (70%)	0.95

*p‐*value between early PHIV and standard PHIV. ART, antiretroviral therapy; Early ART PHIV, PHIV children who early initiated antiretroviral therapy within three months of age; NNRTI, non‐nucleoside reverse transcriptase inhibitor; PI; protease inhibitor; Standard ART PHIV, PHIV children who initiated antiretroviral therapy within three to twelve months of age.

The characteristics of parents, primary caregivers and the family socio‐economic status are shown in Table 3. Primary caregivers’ age was not different among groups. One third (30% to 39%) of primary caregivers were not the biological parents; most of these were grandparents. The primary reasons for transfer of care were work burdens among biological parents and parents in single‐parent households. Most primary caregivers had some secondary school education, at grade nine level. Overall income was 84% and 62% lower than the average Thai income per family (<25,000 Baht/month or <800 USD/month) in the PHIV and the PHEU group respectively.

**Table 3:** Characteristics of parents, primary caregivers and socio‐economic status

Characteristic	PHEU (n = 100)	PHIV (n = 50)	Early ART PHIV (n = 27)	Standard ART PHIV (n = 23)	*p* value^a^	*p* value^b^
Parents						
Mother age at birth of infants, years, median (IQR)	31 (26 to 35)	25 (20 to 32)	27 (20 to 31)	25 (21 to 33)	0.001	0.004
Father age at birth of infants, years, median (IQR)	34 (29 to 39)	29 (22 to 37)	27 (22 to 35)	32 (22 to 37)	0.003	0.007
Primary caregivers, n (%)						
Parents	69 (69%)	33 (66%)	19 (70%)	14 (61%)	0.76	0.64
Grandparents	25 (25%)	13 (26%)	7 (26%)	6 (26%)		
Other relatives	6 (6%)	4 (8%)	11 (4%)	3 (13%)		
Primary caregiver age, years	38 (33 to 44)	36 (37 to 46)	31 (29 to 45)	38 (25 to 46)	0.22	0.36
Duration of primary caregiver education, years	9 (6 to 13)	9 (6 to 12)	9 (6 to 9)	9 (6 to 12)	0.22	0.37
Primary caregiver depression at enrolment	18 (18%)	7 (14%)	4 (15%)	3 (13%)	0.54	0.90
Primary caregiver depression at 48‐week visit	8 (8%)	11 (22%)	7 (26%)	4 (17%)	0.07	0.04
Income per family, Baht/month						
<10,000	16 (16%)	21 (42%)	12 (44%)	9 (39%)	0.001	0.005
10,000 to 25000	46 (46%)	21 (42%)	11 (41%)	10 (44%)		
>25,000	38 (38%)	8 (16%)	4 (15%)	4 (17%)		

Early ART PHIV, PHIV children who early initiated antiretroviral therapy within three months of age; PHEU; perinatally HIV‐exposed uninfected children; PHIV, perinatally HIV‐infected children; Standard ART PHIV, PHIV children who initiated antiretroviral therapy within three to twelve months of age.

^a^ *p*‐value between PHEU and PHIV; ^b^ *p‐*value among PHEU, early PHIV and standard PHIV.

### 3.2 Neurodevelopmental outcomes by Mullen Scales of Early Learning

#### 3.2.1 Prevalence of global developmental impairment and trajectory pattern

The prevalence of overall GDI was 18% (95% CI 11 to 27) and 32% (95% CI 20 to 47) in PHEU and PHIV children respectively (*p *=* *0.06). For the subgroup analysis, the prevalence of overall GDI was 22% (95% CI 9 to 42) in early ART PHIV and 44% (95% CI 23 to 66) in standard ART PHIV children (Table 4).There were no significant differences in the rate of overall GDI in early ART PHIV compared to the PHEU group, at enrolment and at 48‐week visit (*p *=* *0.62, *p *=* *0.79 and *p *=* *0.70 respectively). PHIV children with standard ART had a higher prevalence of overall GDI compared to PHEU children (*p *=* *0.01), specifically only at study enrolment (*p *=* *0.009). The rate of GDI in the standard ART group declined at 48‐week visit and was comparable to PHEU children (*p *=* *0.23).

**Table 4:** Prevalence of global developmental impairment by Mullen Scales of Early Learning among groups

	Overall (n = 150)		Week 0 (n = 150)		Week 48 (n = 147)	
	% (95% CI)	*p* value	% (95% CI)	*p* value	% (95% CI)	*p* value
PHEU	18 (11 to 27)	Reference	9 (4 to 16)	Reference	16 (9 to 24)	Reference
PHIV	32 (20 to 47)	0.06	18 (9 to 31)	0.12	22 (12 to 36)	0.37
Early ART PHIV	22 (9 to 42)	0.62	7 (1 to 24)	0.79	19 (6 to 38)	0.70
Standard ART PHIV	44 (23 to 66)	0.01	30 (13 to 53)	0.009	26 (10 to 48)	0.23

Global developmental impairment defined as Early Learning Composite (ELC) score by ≤70, ELC score included all subscales except for the gross motor. Early ART PHIV; PHIV children who early initiated antiretroviral therapy within three months of age; PHEU, perinatally HIV‐exposed uninfected children; PHIV, perinatally HIV‐infected children; Standard ART PHIV, PHIV children who initiated antiretroviral therapy within three to twelve months of age.

**Figure 1:**
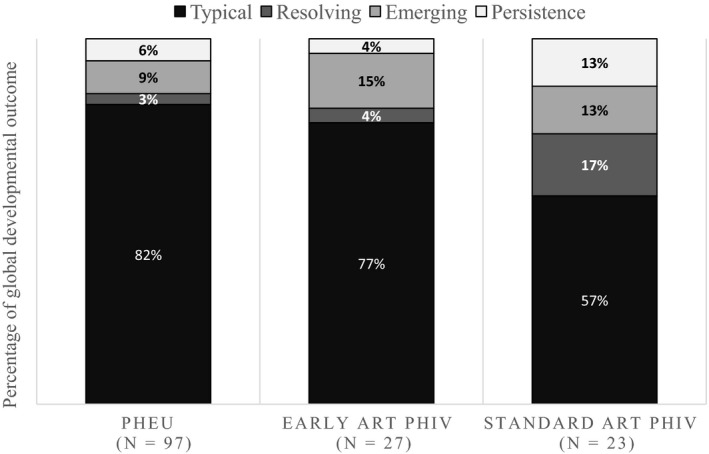
Trajectory patterns of global developmental outcome by ELC score among groups (Early ART PHIV, PHIV children who early initiated antiretroviral therapy within three months of age; ELC, early learning composite; PHEU, perinatally HIV‐exposed uninfected children; PHIV, perinatally HIV‐infected children; Standard ART PHIV, PHIV children who initiated antiretroviral therapy within three to twelve months of age; typical = ELC > 70 at week 0 and week 48, Persistence = ELC ≤ 70 at week 0 and week 48, Resolving = ELC ≤ 70 at week 0, but ELC > 70 at week 48, Emerging = ELC > 70 at week 0, but ELC ≤ 70 at week 48).

The trajectory pattern of global developmental outcome is shown in Figure 1. Typical development was reported among 82%, 77% and 57% in PHEU, early ART PHIV and standard ART PHIV children respectively (PHEU vs. early ART PHIV group, *p *=* *0.75; PHEU vs. standard ART PHIV group, *p *=* *0.02). Four (17%) PHIV children with standard ART had resolving GDI at the 48‐week visit as did one (4%) early PHIV child and 3 (3%) PHEU children. PHIV children had a higher rate of emerging GDI when compared with PHEU children (13% to 15% vs. 9%). Three (13%) standard PHIV children had persistent GDI, whereas one (4%) early ART and 6 (6%) PHEU demonstrated persistent GDI pattern.

#### 3.2.2 Prevalence of individual domain impairment

Prevalence of individual domain impairment is shown in Table 5. Prevalence of impairment in all individual domains among early ART PHIV was comparable to PHEU children. In contrast, prevalence of gross motor impairment was higher in the standard ART PHIV children compared to the PHEU group at enrolment (44% vs. 19%, *p *=* *0.04) but was comparable at the 48‐week visit (25% vs. 19%, *p *=* *0.69). Prevalence rates of fine motor, visual reception, receptive and expressive language impairment were not significantly different in standard ART PHIV children when compared with PHEU children. There were no children with standard ART PHIV who had receptive language impairment at week 48.

**Table 5:** Prevalence of each domain impairment by Mullen Scales of Early Learning among groups

	PHEU % (95% CI)	Early ART PHIV % (95% CI)	*p* value	Standard ART PHIV % (95% CI)	*p* value
Gross motor impairment (GM developmental quotient ≤70)					
Overall	27 (17 to 38)^a^	25 (10 to 47)^b^	0.88	50 (25 to 75)^c^	0.07
Enrollment	19 (11 to 29)	13 (3 to 32)	0.47	44 (20 to 70)	0.04
48‐week visit	19 (9 to 32)	18 (4 to 43)	0.91	25 (3 to 65)	0.69
Fine motor impairment (T score ≤30)^d^					
Overall	23 (15 to 32)	19 (6 to 38)	0.95	30 (13 to 53)	0.95
Enrollment	8 (4 to 15)	7 (1 to 24)	0.92	17 (5 to 39)	0.65
48‐week visit	19 (11 to 28)	15 (4 to 34)	0.18	26 (10 to 48)	0.42
Visual reception impairment (T score ≤30)^d^					
Overall	23 (15 to 33)	16 (6 to 38)	0.62	30 (13 to 53)	0.46
Enrollment	8 (4 to 15)	7 (1 to 24)	0.92	22 (8 to 44)	0.06
48‐week visit	19 (11 to 28)	15 (4 to 34)	0.65	22 (8 to 44)	0.73
Receptive language impairment (T score ≤30)^d^					
Overall	18 (11 to 27)	19 (6 to 38)	0.95	17 (5 to 39)	0.95
Enrollment	15 (9 to 24)	11 (2 to 29)	0.61	17 (5 to 39)	0.78
48‐week visit	8 (4 to 16)	17 (5 to 39)	0.78	0 (0 to 15)	NA
Expressive language impairment (T score ≤30)^d^					
Overall	34 (25 to 44)	22 (9 to 42)	0.25	35 (16 to 57)	0.94
Enrollment	19 (12 to 28)	7 (1 to 24)	0.17	26 (10 to 48)	0.45
48‐week visit	29 (20 to 39)	22 (9 to 42)	0.50	26 (10 to 48)	0.79

*p*‐value when compared with PHEU children, gross motor developmental quotient derived from age equivalent score divided by actual age and multiply with 100 (a mean of 100 and SD of 15). Early ART PHIV, PHIV children who early initiated antiretroviral therapy within three months of age; PHEU, perinatally HIV‐exposed uninfected children; PHIV, perinatally HIV‐infected children; Standard ART PHIV, children who initiated antiretroviral therapy within three to twelve months of age.

^a^n for PHEU = 79, 79 and 53 at overall, enrolment, and 48‐week visit; ^b^n for early ART PHIV = 24, 24 and 18 at overall, enrolment, and 48‐week visit; ^c^n for standard ART PHIV = 16, 16 and 8 at overall, enrolment, and 48‐week visit; ^d^T‐score of other domains derived from raw scores with a mean of 50 and SD of 15, n for PHEU children = 100, 100 and 97 at overall, enrolment, and 48‐week visit, n for early ART PHIV = 27 and n for standard ART PHIV = 23 at all visits.

#### 3.2.3 Developmental scores (ELC and each domain score)

**Figure 2:**
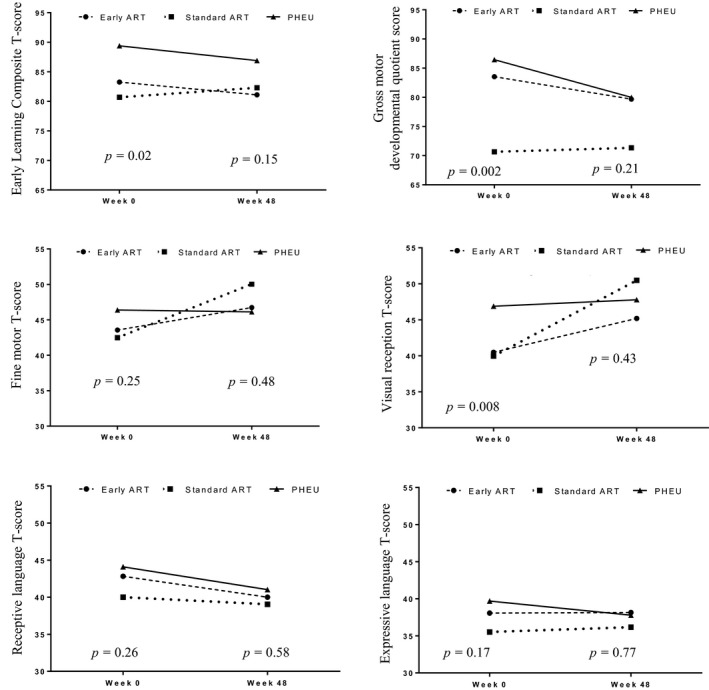
Comparison of Mullen Scales of Early Learning overtime among groups (Early ART PHIV, PHIV children who early initiated antiretroviral therapy within three months of age; ELC, early learning composite; PHEU, perinatally HIV‐exposed uninfected children; PHIV, perinatally HIV‐infected children; Standard ART PHIV, PHIV children who initiated antiretroviral therapy within three to twelve months of age, *p*‐value; compare among three groups).

Comparison of developmental scores among groups of study participants is shown in Table 6 and Figure 2. Mean (SD) ELC scores at enrolment were 90 (16), 83 (11), 81 (19) in PHEU, early ART PHIV and standard ART PHIV children, respectively, with significant differences among three groups (*p *=* *0.02) and between PHEU and standard ART PHIV (*p *=* *0.01). However, no group differences were observed at week 48 among three groups and when compared with PHEU (Mean (SD) 87 (15), 81 (15) and 82 (16) in PHEU, early ART PHIV and standard ART PHIV children, respectively, *p *>* *0.05). Mean ELC score declined overtime in PHEU and early ART PHIV children (mean difference −2.7 (95% CI −5.4 to 0.05 in PHEU and −2.1 (95% CI −8.5 to 4.2) in early ART PHIV group). Mean scores increased in standard ART PHIV children [mean difference 1.6 (95% CI −4.2 to 7.4)).

**Table 6:** Comparison of neurodevelopmental scores by Mullen Scales of Early Learning among groups

	Week 0		Week 48	
	Mean (SD)	*p* value	Mean (SD)	*p* value
Early learning composite score				
PHEU	90 (16)	Ref	87 (15)	Ref
Early ART PHIV	83 (11)	0.05	81 (15)	0.09
Standard ART PHIV	81 (19)	0.01	82 (16)	0.20
Gross motor developmental quotient				
PHEU	87 (16)	Ref	80 (12)	Ref
Early ART PHIV	84 (11)	0.41	80 (11)	0.99
Standard ART PHIV	71 (16)	<0.001	71 (23)	0.08
Fine motor T‐score				
PHEU	47 (12)	Ref	46 (14)	Ref
Early ART PHIV	44 (10)	0.21	47 (14)	0.84
Standard ART PHIV	43 (13)	0.12	50 (16)	0.23
Visual reception T‐score				
PHEU	47 (13)	Ref	48 (14)	Ref
Early ART PHIV	41 (9)	0.01	45 (14)	0.41
Standard ART PHIV	40 (13)	0.01	51 (16)	0.42
Receptive language T‐score				
PHEU	44 (11)	Ref	41 (9)	Ref
Early ART PHIV	43 (8)	0.54	40 (8)	0.59
Standard ART PHIV	40 (11)	0.09	39 (5)	0.32
Expressive language T‐score				
PHEU	40 (10)	Ref	38 (11)	Ref
Early ART PHIV	38 (7)	0.44	38 (10)	0.87
Standard ART PHIV	36 (9)	0.06	36 (11)	0.51

Early learning composite score derived from total scores of all subscales except for gross motor with a mean of 100 and SD of 15, gross motor developmental quotient with a mean of 100 and SD of 15 and other T‐scores derived from raw scores with a mean of 50 and SD of 15. PHEU, perinatally HIV‐exposed uninfected children; PHIV, perinatally HIV infected children; Early ART PHIV, PHIV children who early initiated antiretroviral therapy within three months of age; Standard ART PHIV, PHIV children who initiated antiretroviral therapy within three to twelve months of age.

Standard ART PHIV children had significantly lower gross motor developmental quotient when compared with PHEU children at enrolment (*p *<* *0.001): however, no difference was observed at the 48‐week visit. Early ART PHIV children had comparable performances in all domains when compared with PHEU children, except for lower visual reception T‐score at enrolment (mean (SD) 41 (9) vs. 47 (13), *p *=* *0.01). On the contrary, standard ART PHIV children had lower scores in all domains with significant differences in gross motor and visual reception domain when compared with PHEU children at the enrolment visit. However, no differences were observed at the 48‐week visit. Standard ART PHIV children showed a significant increase in fine motor T‐score (mean difference 7.6 (95% CI 1.4 to 13.7) and visual reception T score (mean difference 10.5 (95% CI 3.4 to 17.6) from week 0 to week 48.

### 3.3 Predictors of changing ELC score

Regarding predictors of declines in ELC scores, non‐attendance in nursery school remained a significant factor (adjusted coefficient −3.8, 95% CI: −6.1 to −1.6, *p *=* *0.001), after adjustment for relevant child, caregiver and family covariates specified in the methods. (Table 7).

**Table 7:** Predictors of changes in Early Learning Composite (ELC) score

	Univariate			Multivariate		
	Coeff	95% CI	*p* value	Coeff	95% CI	*p* value
Group						
PHEU	1	Ref				
Early ART PHIV	−1.1	−4.0 to 1.8	0.47			
Standard ART PHIV	0.3	−2.9 to 3.3	0.88			
Age initiated ART^a^	0.7	−0.2 to 1.5	0.12			
Duration of ART, months^a^	0.1	−0.1 to 0.2	0.61			
HIV‐RNA ≥ 200 copies/mL^a^	−3.3	−7.4 to 0.7	0.11			
No attendance to nursery school	−3.6	−5.7 to −1.4	0.001	−3.8	−6.1 to −1.6	0.001
Mother age, years	0.1	0.0 to 0.3	0.13			
Duration of mother's education, years	0.3	0.0 to 0.6	0.04	0.1	−0.2 to 0.4	0.43
Father age, years	0.1	0.0 to 0.2	0.11			
Duration of father's education, years	0.2	−0.1 to 0.6	0.13			
Primary caregiver not biological parents	0.9	−1.4 to 3.2	0.46			
Primary caregiver age, years	0.1	0.1 to 0.2	0.04	0.1	−0.5 to 0.2	0.31
Duration of primary caregiver's education, years	0.1	−0.2 to 0.4	0.46			
Primary caregiver depression score	−0.2	−0.5 to 0.1	0.26			
Income per family <10,000 Baht/month	−2.9	−5.4 to −0.4	0.02	−2.2	−4.9 to 0.5	0.12

The following variables were also included in the model but were not significant at 0.05 level and are not shown: children gender, birth weight, prematurity, children nutritional status, children anthropometric status, maternal drug use history and parenting style. Early ART PHIV, PHIV children who early initiated antiretroviral therapy within three months of age; PHEU, perinatally HIV‐exposed uninfected children; PHIV, perinatally HIV infected children; Standard ART PHIV, PHIV children who initiated antiretroviral therapy within three to twelve months of age.

a These variables were derived data from PHIV group.

## 4 Discussion

Our study of young children with PHIV in Thailand revealed a protective effect of early ART initiation on early global developmental functioning, as measured by the MSEL. Children treated within the first three months of life demonstrated relatively low and comparable rates of GDI to PHEU children at study entry. In contrast, children with later ART initiation, at three to twelve months of age, demonstrated higher prevalence of developmental impairment overall and gross motor impairment at study entry compared to PHEU children. Of interest, however, follow‐up developmental evaluations one year after study entry revealed similar rates of GDI among PHEU children and both groups of PHIV children who were treated within the first year of life. These results reinforce the benefits of ART to reduce developmental risk among children with PHIV and identify variable impact of early and later ART initiation in young children with PHIV.

Earlier studies revealed variable rates of GDI among PHIV children. A recent meta‐analysis of neurodevelopment in PHIV and PHEU young children reported developmental impairment (−2 SD below the mean on the Bayley Scales of Infant Development) among 21% to 35% of children; however, most of these studies did not indicate onset of ART 29. Our study revealed that 32% of PHIV children had developmental impairment overall, yet impairment was significantly less likely among children with early initiation of ART (22%) compared to those with later initiation of ART PHIV (44%). With respect to trajectory patterns, we observed that PHIV children with early ART demonstrated a higher rate of typical developmental functioning than PHIV children with standard ART (77% vs. 57%). As such, this study highlighted the importance of ART initiation within three months of age to mitigate deleterious effects of HIV on early neurodevelopmental outcomes, as previously reported in South African studies 17, 30.

Both our results and those of the CHER study found that PHIV children with standard ART initiation demonstrate developmental catch‐up as they age. We initially hypothesized that longer duration of ART or viral suppression status may be associated with better developmental outcomes, yet the secondary analysis did not identify these associations. Instead, we speculated that early, appropriate developmental stimulation may be key to improving development 31, 32.Children with PHIV have frequent regularly scheduled check‐ups with their paediatricians in order to access ART every one to three months; such frequent interactions with healthcare providers provide increased opportunities for parents to ask for and receive advices regarding their child's neurodevelopmental functioning. Such advices may assist parents in accessing specific therapies and/or providing appropriate stimulation, play and teaching with their child at home, thus increasing opportunities for developmental growth. Children with HEU, in contrast, have lengthy intervals between paediatric check‐ups, with appointments typically scheduled every six to twelve months at ages >2 years. Therefore, opportunities for parental support from healthcare providers regarding child stimulation/education may be reduced for these families compared to children with HIV. Besides, in our study, developmental paediatricians routinely provided suggestions to caregivers regarding how to support and stimulate their children; they also referred children to therapeutic services if delays were identified. Importantly, we noted that 75% of children with resolving developmental impairment were attending nursery school during the study. The enriched social and educational environment of nursery school is likely beneficial for PHIV children.

Prior studies of young children with PHIV described lower mean developmental scores including motor scores among PHIV children compared to PHEU 4, 33. Our results indicate that only PHIV children with later ART initiation, at three to twelve months of age, had significantly lower general developmental score than PHEU at study enrolment. These results are consistent with those of the CHER trial in which early ART PHIV did not differ in general neurodevelopmental scores when compared with PHEU children, whereas those with deferred ART had lower scores in early life that later resolved 17, 18. In addition, trials in South Africa and Kenya showed that PHIV infants with ART initiation at median age of four months old demonstrated delayed developmental milestones in the first few years of life compared to healthy HIV‐unexposed children 34, 35. Such findings underscore a narrow window of time to optimally initiate ART during early infancy in order to preserve early healthy neurodevelopmental outcomes in PHIV individuals 15.

In contrast to findings of the CHER study, gross motor functioning was not significantly delayed in early ART PHIV children compared to PHEU children in our study 17. Nevertheless, the gross motor domain was still the major domain of impairment in PHIV children with standard ART initiation in early life, with subsequent improvement at age four to five years old. Regardless of the timing of ART initiation, we also observed delays in visual reception at study entry among PHIV children compared to PHEU children but there was improvement at the follow‐up week‐48‐visit. The CHER study detected visual reception impairment at age five years in both the early and deferred ART group. It is interesting to note that fine motor and visual reception performance was significantly increased in the standard ART PHIV group and even higher than the early ART and the PHEU group at the 48‐week visit. These observations suggest that neurodevelopment is very dynamic and that the standard ART PHIV group had potential to improve their neurodevelopmental functioning over time under enriched environments. Nonetheless, this should be interpreted with caution due to a relatively small number of children and the short period of follow‐up. Our results did not indicate deficits in language functioning among PHIV children as in prior studies 4, 11, 28, 35. This may be due to relatively lower expressive language scores in all groups that might reflect our participants’ impoverished language environment.

We found that non‐attendance at nursery school was associated with neurodevelopmental decline 16. In Thailand, children attend nursery or daycare at two to four years old and often begin preschool/kindergarten at three to six years old. Children are taught basic skills and knowledge through creative play and social interaction. Both home‐based and school‐based interventions could improve early childhood neurodevelopmental functioning by providing increased opportunities for developmental stimulation and learning 7, 36, 37, 38, 39, 40, 41, 42. Even though growth parameters were significantly lower in PHIV children, they were not associated with developmental outcomes. Overall there was a low rate of stunting, underweight and microcephaly among the participants 16, 43. We also observed a lower rate of caregiver depression in our PHIV group than observed in African studies. 44, 45. No association was found between caregiver depression and developmental outcomes in our study, although such associations have been observed in earlier studies 46, 47.

The strengths of this study include careful documentation of the timing of ART initiation, the longitudinal nature of the study, excellent retention of both study groups and consideration of multiple demographic and psychosocial factors that potentially influence child developmental outcomes. Moreover, all primary caregivers were provided appropriate counselling and guidance on how to maximize their children's development during study participation, and children with significant developmental problems were referred to therapeutic services.

However, there were several limitations to acknowledge. First, this study has no HIV‐unexposed uninfected children as a comparison group, due to our concerns about differences in socio‐economic backgrounds among Thai HUU children and PHIV and PHEU children as well as the known impact of socio‐economic backgrounds upon development, as previously reported in the PREDICT study 13. Few healthy children (0.4% to 2.6%) in middle‐to‐high socio‐economic status families in Thailand demonstrate GDI, as assessed by MSEL 48. For this reason and others, we enrolled only PHEU children as our comparison group. In addition, despite attempts to enrol PHEU with similar backgrounds as PHIV children, there were significant differences in family characteristics and socio‐economic status between groups. This could compromise our ability to understand the effect of HIV and ART exposure on child development. However, the potential confounding effects were controlled in part by the multivariate analysis. Second, potential bias of recruitment of PHIV group could be introduced since primary caregivers of PHIV children were invited to participate; these primary caregivers may already have concerns about developmental difficulties of their children or other HIV‐related health problems. However, most PHIV children were asymptomatic and the rate of viral suppression was similar to the overall Thai data with a range of 68% to 70% viral suppression 49. Third, the median age of assessment was different for early ART versus standard ART children. In Thailand, the schedule of PCR HIV for HIV diagnosis in infants is at one, two and four months and the early infant diagnosis by dried blood spot at birth provided since 2010 21. Most of standard ART PHIV children were born during 2012 to 2014 and the early ART PHIV children were born during 2014 to 2016, due to improvement of early diagnosis. However, the MSEL test was administered as appropriate for each child's age and assigned scores were based on MSEL norms. Finally, the brief follow‐up may not capture all differences in developmental outcomes among groups, especially during dynamic developmental periods early in life that could be affected by unmeasured factors.

## 5 Conclusions

Rates of GDI in PHIV children who initiated ART within three months of age were similar to PHEU children and significantly lower than children who were treated at three to twelve months of age. Developmental improvement was observed among those with late ART after one year of follow‐up. Non‐attendance at nursery school was associated with poor developmental scores over time. This study emphasizes that missed opportunities for early initiated ART may lead to early developmental challenges. Although ongoing follow‐up of this cohort will further inform our understanding of the long‐term benefits of early ART initiation and other factors associated with appropriate developmental outcomes, it remains essential to establish an effective system to ensure early diagnosis and early treatment of PHIV infants. In addition, these vulnerable children with PHIV require close monitoring of attainment of developmental milestones as well as early developmental stimulation.

## Competing interests

JA has received honoraria for participating in advisory meetings for AbbVie, Roche, Gilead, Merck and ViiV Healthcare. PT and NJ have received partial research support from Biogen, Inc. (Boston, USA) on topics unrelated to the research in this manuscript. The other authors declare that they have no conflicts of interest.

## Authors’ contributions

WJ, TP, WC, MV, NJ, PT, KM, JA and CP contributed to the study concept and design. JS and TT were the study coordinator. WJ, WC, TP and KM contributed to analysis and interpretation of the data. SP was statisticians. WJ wrote the first draft of the paper and all co‐authors contributed to interpreting the findings and revised the manuscript. All authors read and approved the final manuscript.
